# Single-cell profiling delineates the tumor microenvironment and immunological networks in patient-derived uterine leiomyosarcoma

**DOI:** 10.3389/fimmu.2025.1653096

**Published:** 2025-08-29

**Authors:** Yi Guo, Dongsheng Shen, Yuhang Xiao, Chenghao Wu, Meiyi Chen, Lina Yang, Huaifang Li, Xiaowen Tong, Rujun Chen, Fang Li

**Affiliations:** ^1^ Department of Obstetrics and Gynecology, Shanghai East Hospital, School of Medicine, Tongji University, Shanghai, China; ^2^ Department of Laboratory Medicine, Shanghai Tongji Hospital, School of Medicine, Tongji University, Shanghai, China; ^3^ Department of Obstetrics and Gynecology, Shanghai Tongji Hospital, School of Medicine, Tongji University, Shanghai, China; ^4^ Department of Obstetrics and Gynecology, International Peace Maternal and Child Health Hospital of China Welfare Society, Shanghai Jiaotong University, Shanghai, China; ^5^ Cancer Institute, Fudan University Shanghai Cancer Center, Shanghai, China; ^6^ Department of Oncology, Shanghai Medical School, Fudan University, Shanghai, China; ^7^ Department of Gynecology and Obstetrics, Shanghai Fifth People’s Hospital, Fudan University, Shanghai, China

**Keywords:** uterine leiomyosarcoma, metastasis, tumor microenvironments, single-cell RNA sequencing, prognosis, T cell, macrophage

## Abstract

**Background:**

Uterine leiomyosarcoma (ULSA) is a highly aggressive gynecologic malignancy characterized by early metastasis, profound immunosuppression, and resistance to conventional therapies, including immune checkpoint blockade (ICB). The intricate tumor microenvironment (TME) and cellular heterogeneity driving its progression and therapy resistance remain poorly defined.

**Methods:**

We performed single-cell RNA sequencing (scRNA-seq) on metastatic lesions (pelvic cavity, rectum, peritoneum, bladder) from a treatment-naïve ULSA patient and compared them to normal uterine myometrium, MMM (n=5). Integrated analyses included cellular composition mapping, copy number variation (CNV) assessment, pseudotemporal trajectory reconstruction, cell-cell communication inference, functional enrichment, and validation via multiplex immunofluorescence (mpIF). Survival correlations were assessed using the TCGA-SARC cohort.

**Results:**

In this study, the main finding is that the tumor microenvironment (TME) has a strong immunosuppressive effect. Firstly, its characteristic is exhausted CD8^+^T cells. This study found that as time progresses, the initial cell markers (CCR7, MAL) gradually disappear, while the exhaustion markers (LAG3, HAVCR2, TIGIT) are enriched. This is associated with poor prognosis. Secondly, the M2-polarized macrophages are mainly composed of M2-like tumor-associated macrophages (TAMs) with tumor-promoting characteristics (CD163, FTH1, FTL, TIMP1), and there is a polarization from M1 to M2. Finally, the immature, tumor-promoting N2 neutrophils (CD15^+^EDARADD^+^) enriched in the metastatic foci are associated with poor prognosis. The cell communication involves the interaction of MIF-(CD74+CD44) between T/B cells, as well as the role of the CXCL8 signaling axis in promoting angiogenesis, TAM polarization, and immunosuppression.

**Conclusion:**

For the first time, a comprehensive single-cell map of ULSA was constructed, depicting a metastasis-susceptible cell subset (U11-EDARADD) and an extremely immunosuppressed tumor microenvironment dominated by depleted CD8^+^T cells, M2 macrophages and N2 neutrophils. These features shed light on the underlying mechanisms of chemotherapy resistance and immunotherapy failure. The biomarkers identified here (EDARADD, CLDN10, TMIGD2) as well as the dysregulated pathways (TGF-β, angiogenesis, MIF signaling) provide possible targets for future development of combined immunotherapy strategies against this deadly disease.

## 1 Introduction

Uterine leiomyosarcoma (ULSA), representing approximately 3%–9% of uterine malignancies, is a highly aggressive yet rare gynecological cancer that poses significant diagnostic and therapeutic challenges (1–3). Most leiomyosarcomas display high-grade histology, which correlates with poor prognoses (4). The current standard treatment for early-stage ULSA is hysterectomy; however, recurrence rates remain high (53%–71%), often with early hematogenous dissemination (5). In advanced or recurrent disease, first-line systemic therapy relies on doxorubicin-based regimens, yet outcomes remain suboptimal, demonstrating overall response rates (OR) of merely 25%–38% and a median overall survival (OS) of less than two years (5, 6).

However, advancing targeted therapies is impeded by insufficient knowledge of the immune landscape (7–9). ULSA exhibits an immunosuppressive microenvironment that typically confers resistance to immunotherapies. Although immune checkpoint blockade (ICB) has demonstrated effective in other treatment-refractory malignancies, clinical studies indicate minimal response in ULSA (10). A phase 2 single-center trial found that nivolumab-mediated PD-1 inhibition conferred no clinical benefit in advanced ULSA patients (11). George S et al. implicated PTEN loss potentially contributes to ICB resistance in metastatic ULSA (12). De Wispelaere W et al. suggested that dysregulated PI3K/mTOR signaling may further reinforce an immunosuppressive tumor microenvironment (TME), possibly explaining ICB resistance in ULSA (13). In the multicenter phase 3 LMS-04 trial, first-line doxorubicin combined with trabectedin significantly improved progression-free survival (PFS) compared to doxorubicin alone in metastatic or advanced leiomyosarcoma (14). However, the increased toxicity of combination regimens necessitates careful patient selection (14). Despite these advances, the genetic drivers and signaling pathways underlying ULSA pathogenesis remain poorly defined, underscoring the need for further mechanistic investigation.

Recent advances in single-cell sequencing have enabled high-resolution characterization of tumor heterogeneity (15, 16), the tumor microenvironment (TME), and molecular mechanisms driving oncogenesis and progression (17–19). Here, we present a treatment-naïve metastatic ULSA patient who underwent three cytoreductive surgeries over four years, with metastatic lesions collected from the pelvic cavity, rectum, abdominal wall, and bladder for single-cell profiling. By integrating TCGA database analyses, we systematically investigated the immune microenvironment within ULSA metastatic foci to identify potential therapeutic vulnerabilities. Although prior studies have cataloged ULSA transcriptomic and genomic alterations (20–22), this study provides the first single-cell resolution atlas of ULSA pathophysiology. Our findings advance the understanding of ULSA TME biology and may offer novel insights for developing targeted treatment and prevention strategies.

## 2 Materials and methods

### 2.1 Human studies statement

This investigation received approval after review by the Institutional Ethics Committee of Shanghai Tongji Hospital, School of Medicine, Tongji University, Shanghai, China (No. K-W-2024-016). Tumor specimens were obtained from a ULSA patient following provision of written informed consent. Freshly resected lesions were immediately placed in specialized tissue preservation medium on ice and prepared for immediate transfer.

### 2.2 Sample preparation

Surgical resection under aseptic conditions was performed to obtain ULSA tumor specimens from the diagnosed patient and normal myometrial tissues from five age-matched (within 5 years), premenopausal patients undergoing hysterectomy for benign conditions. To ensure anatomical consistency, all tissues were harvested from the deep myometrial layer, avoiding endometrial contamination. Immediately after excision, specimens were rinsed with ice-cold sterile PBS to remove debris and preserve viability. Using sterile instruments, tissues were dissected into 1–5 mm fragments. Within 24 hours, fragments were enzymatically digested in a pre-warmed solution with 5 mM EDTA, 1 mM DTT, 15 mM HEPES in PBS with 10% heat-inactivated FBS at 37°C. After initial digestion, samples were washed twice with PBS to remove residual enzymes. Secondary digestion was performed using 0.38 mg/mL collagenase VIII and 0.1 mg/mL DNase I in complete DMEM supplemented with 100 U/mL penicillin and 100 μg/mL streptomycin. Gentle pipetting at 10-minute intervals over 60 minutes ensured efficient dissociation while minimizing cell damage. The resulting suspension was filtered through a 100-μm nylon mesh via gravity flow to prevent shear stress. The filtrate was centrifuged at 300 rpm for 5 minutes, and the pellet was resuspended in complete DMEM. FBS (Thermo Fisher Scientific, Uppsala, Sweden) and enzymes (Sigma-Aldrich, Steinheim, Germany) were quality-controlled for lot consistency and activity. Cell viability and counts were assessed, and only samples meeting predefined thresholds were processed for scRNA-seq.

### 2.3 scRNA sequencing

Single-cell gel bead manufacturing was performed in strict accordance with the 10x Genomics Chromium 3’ v3 kit protocol (10x Genomics, Pleasanton, CA). For sequencing library preparation, both single-cell RNA libraries and TCR V(D)J libraries were constructed following standardized procedures. Sequencing was carried out on an Illumina NovaSeq 6000 system using 150 bp paired-end reads (PE150). To ensure data reproducibility and quality, all manufacturer-recommended protocols were rigorously followed, including pre-use validation of instruments and reagents. Additionally, laboratory ambient conditions, particularly temperature and humidity, were maintained within specified tolerances to minimize technical variability.

### 2.4 Data screening and quality control

Processed single-cell sequencing data were analyzed using R (v4.3.2). Prior to quality control (QC), potential doublets—artificial cell aggregates resulting from multiple cell encapsulations—were identified and removed using DoubletFinder (v2.0.3). Doublet scores were computed based on gene expression profiles, with thresholds optimized according to expected doublet rates derived from cell loading densities. Cells exceeding the calculated threshold were excluded to ensure each analyzed unit represented a single cell. Quality control procedures implemented via Seurat (23) (v5.1.0) in R (v4.3.2) involved excluding cells exhibiting: fewer than 200 detected genes, mitochondrial gene content exceeding 10%, or total UMI counts below 500. This filtration step eliminated potentially compromised cells that might bias subsequent analyses. Following filtration, data normalization was performed using Seurat’s NormalizeData function. Genes demonstrating high biological heterogeneity were then identified through the FindVariableFeatures function. To address technical variability across different sequencing batches, batch effect correction was applied using the Harmony package (v0.1.1) with the RunHarmony function, which integrates batch-corrected dimensions into the downstream analysis workflow. This step adjusts for systematic differences between batches while preserving genuine biological variation, as validated by reduced batch-specific clustering in post-correction visualization.

### 2.5 Dimensionality reduction, clustering and cell type identification

Dimensionality reduction was achieved via Principal Component Analysis (PCA) using the RunPCA function on the harmony-corrected data, with the analysis restricted to the top 2000 highly variable genes identified in Section 2.4 to focus on biologically meaningful variation. The RunPCA function was implemented with the parameter npcs = 50 to generate a sufficient number of principal components (PCs) for downstream analysis. Optimal principal component quantity was established through the elbow method, with the inflection point on scree plots typically identified between PCs 15–20 based on the stabilization of explained variance. Subsequent clustering utilized the FindClusters function with the Louvain algorithm (algorithm = 1), where the resolution parameter was optimized through iterative testing across the range 0.4–1.2. The final resolution (0.8) was selected based on two criteria: (1) consistent separation of clusters with distinct marker gene expression and (2) minimal over-clustering of biologically homogeneous populations, as validated by silhouette width analysis. Cell type annotation was performed using SingleR (v2.8.0). Cluster-level identity assignment was conducted in method = “cluster” mode, which aggregates expression profiles across all cells within a cluster. Differential gene expression analysis was then applied (de.method = “wilcox”) to identify statistically significant marker genes. Annotation confidence thresholds were stringently set at >0.7 (SingleR pruned.labels score) to ensure robust transcriptomic matching. Computational assignments underwent manual validation using Seurat’s FeaturePlot and VlnPlot, with expert investigators evaluating cluster distributions. Marker gene expression was quantified (AverageExpression, assay=“RNA”) to verify cluster-specific enrichment (log2FC >1.5, adj.p <0.01), cross-referenced against established signatures. Discrepancies triggered iterative re-analysis, including clustering resolution adjustments (± 0.1 increments) and PCA re-runs with refined variable gene sets, until alignment with canonical phenotypes was achieved.

### 2.6 Copy number variation analysis

Copy number variations were evaluated in ULSA using the inferCNV R package version 1.20.0 to assess genomic instability, with uterine myometrium, MMM serving as the reference group. The “infercnv::run” function was applied with key parameters including a 0.1 expression cutoff to filter lowly expressed genes, group-based clustering to avoid confounding effects, denoising to reduce technical noise, and a six-state Hidden Markov Model for robust CNV prediction. Processed expression matrices were exported for downstream analysis (16). A 101-gene sliding window approach was used for signal smoothing. Comparative analysis revealed consistent amplification and deletion patterns in ULSA relative to MMM, demonstrating chromosomal instability and further supporting the malignant nature of this tumor type.

### 2.7 Analysis of differentially expressed genes

Differential gene expression analysis was performed using the Seurat package (v5.1.0) in R. Tumor and normal cell groups were defined based on sample metadata and cell-type annotations, and subsetted from the integrated Seurat object via the subset function (parameter: idents). The FindMarkers function was applied with the following parameters: test.use = “wilcox” for non-parametric group comparisons; logfc.threshold = 0.25 to identify genes with modest expression changes; min.pct = 0.1 to exclude genes expressed in <10% of cells in either group, mitigating low-expression noise; and min.cells.group = 3 to ensure robust group representation. The RNA assay (assay = “RNA”) provided normalized input data, with significance thresholds set at adjusted *P*-value <0.05. Output included gene symbols, average log2 fold changes, expression frequencies per group, raw *P*-values, and Benjamini-Hochberg-adjusted *P*-values, enabling downstream biological interpretation.

### 2.8 Pseudotime analysis

Pseudotemporal trajectory reconstruction was performed using Monocle 2 (v2.34.0) in R (24), beginning with the construction of a CellDataSet object from single-cell RNA sequencing data (25). This process integrated gene expression matrices, cellular metadata, and analytical parameters. Data preprocessing included normalization and feature selection via the preprocessCDS function, followed by dimensionality reduction using the reduceDimension method, which internally applied PCA or tSNE. Cellular pseudotemporal ordering was then inferred through the orderCells function, reconstructing developmental trajectories based on transcriptional dynamics along the inferred paths. Finally, trajectory visualization and transcriptional profiling were achieved using plot_cell_trajectory, enabling chronological interpretation of differentiation states.

### 2.9 Cell-to-cell communication

Cell-cell communication analysis was conducted using the CellChat package version 2.1.0 in R as previously described (26). Single-cell transcriptomic data were preprocessed through integration of cellular annotations with gene expression matrices. The analytical workflow followed four key steps: initial creation of a CellChat object using the createCellChat function, identification of cell-type-specific overexpressed genes and shared upregulated genes through the identifyOverExpressedGenes and identifyOverlappingGenes functions respectively, computation of intercellular communication probabilities via the computeCommunications function based on these gene sets, and rigorous filtering of interactions. Statistical significance was determined using a permutation test-derived p-value threshold of less than 0.05, with additional false discovery rate control set at less than 0.1 through Benjamini-Hochberg adjustment. The resulting interaction networks and their signaling intensities were subsequently visualized using the netplot function, revealing systematic patterns of intercellular crosstalk.

### 2.10 Functional analysis

Transcriptomic data derived from single-cell experiments were analyzed using the irGSEA package (v2.1.5) in R (27, 28). Gene set enrichment analysis (GSEA) was performed via the gsea function, with predefined gene sets curated from standardized databases (29). This method quantified the statistical overrepresentation of functionally annotated gene sets within specific cellular subpopulations or experimental conditions. Results included enrichment scores, adjusted *P*-values, and false discovery rates (FDR), facilitating systematic interpretation of transcriptional patterns in biological contexts.

### 2.11 Hematoxylin and eosin staining

Lesion specimens underwent overnight fixation in 10% formaldehyde for structural preservation. Sequential dehydration through ascending ethanol concentrations (70%, 80%, 90%, 100%) preceded paraffin embedding. Microtome sectioning produced 5 µm-thick tissue slices. Dewaxing occurred through xylene incubation followed by descending ethanol rehydration. Rehydrated sections received hematoxylin application (5 minutes) for nuclear staining, subsequently rinsed under flowing water. Cytoplasmic counterstaining employed eosin immersion (2 minutes). Final processing included ethanol dehydration, xylene clearing, and slide mounting for microscopic evaluation.

### 2.12 Multiplex immunofluorescence

The multiplex immunofluorescence (mpIF) assay was performed according to the protocol established by Cao et al. (30). Formalin-fixed, paraffin-embedded (FFPE) patient specimens were sectioned at 4 μm thickness and subjected to immunofluorescent staining using the following markers: neutrophils (CD15, 1:100 dilution, Thermo Fisher Scientific), tumor cells (SMA, 1:500, Cell Signaling Technology), EDARADD (1:100, Thermo Fisher Scientific), and CLDN10 (1:100, Thermo Fisher Scientific). Primary antibody incubation (30 min) was followed by secondary antibody application (10 min), with nuclear counterstaining using diamidino-2-phenylindole (DAPI, Sigma-Aldrich). Tyramide signal amplification (TSA 570) fluorescence labeling was conducted for 10 min. After TBST washing, slides were immersed in preheated citrate solution and subjected to microwave irradiation (15–20 min) before equilibration to ambient temperature. Digital imaging was performed using the PanoVIEW VS200 slide scanner (Panovue), with biological triplicates ensuring experimental reproducibility.

## 3 Results

### 3.1 Diagnosis and management of a ULSA patient

**Figure 1:**
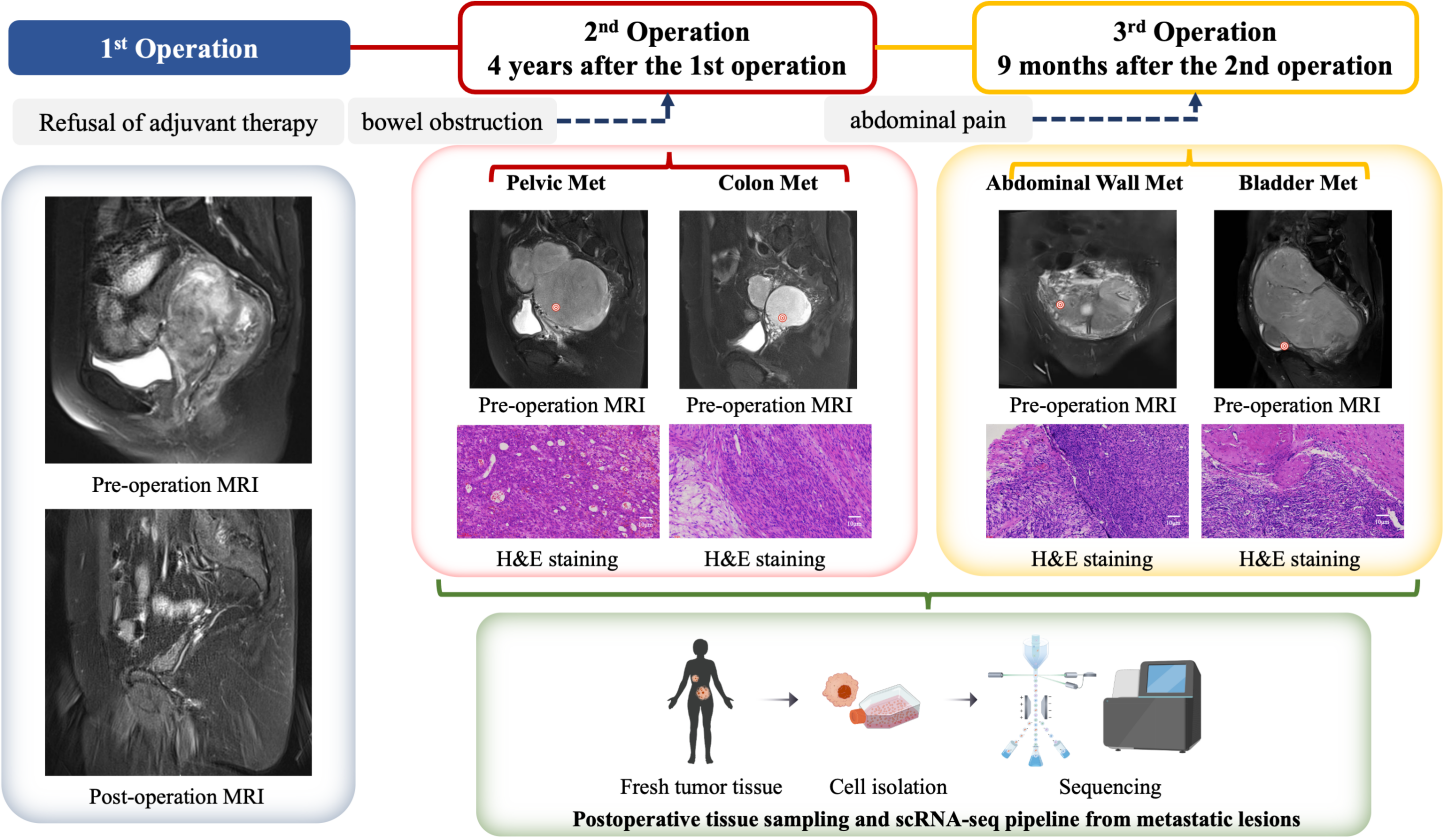
Clinical management algorithm for a ULSA case. The patient initially presented 4 years ago with complaints of lower abdominal pain and a palpable fixed mass in the lower abdomen. Pelvic magnetic resonance imaging (MRI) revealed a large mass located between the uterus and rectum, raising suspicion for a malignant neoplasm, with uterine sarcoma being among the differential diagnoses. An exploratory laparotomy was subsequently performed, during which a total hysterectomy, bilateral salpingo-oophorectomy, and cytoreductive surgery for the pelvic mass were carried out. Postoperative histopathological examination confirmed the diagnosis of uterine leiomyosarcoma (ULMS). The patient declined any form of adjuvant therapy following the surgical intervention. Four years later, the patient returned with metastatic ULSA involving the pelvis and rectum, prompting a second cytoreductive surgery. Despite the recurrence, the patient again refused any adjuvant treatment. Nine months after the second surgery, the patient presented with abdominal wall and bladder metastases, necessitating a third cytoreductive surgery. During the third surgical procedure, metastatic tumor specimens were procured from the pelvic cavity, rectum, peritoneum, and bladder. Single-cell analysis was performed on these specimens, and in conjunction with data from The Cancer Genome Atlas (TCGA) database, we conducted a comprehensive analysis of the immune microenvironment within metastatic lesions of ULSA. This integrated analysis elucidated the genetic characteristics and immunological landscape of the tumor microenvironment (TME) in ULSA.

A 50-year-old patient with uterine leiomyosarcoma was treated at Shanghai Tongji Hospital. The initial presentation 4 years ago included lower abdominal discomfort, and physical examination revealed abdominal distension with a fixed pelvic mass. MRI identified a large uterorectal space-occupying lesion, raising suspicion for malignancy, particularly uterine sarcoma. Metastatic workup (abdominal and thoracic CT) showed no distant involvement. Surgical management comprised cystoscopic bilateral ureteral Double-J stent placement, exploratory laparotomy with total hysterectomy, bilateral salpingo-oophorectomy, and pelvic mass cytoreduction. Intraoperative findings included a uterus of normal dimensions (18 × 15 × 12 cm) with a multicomponent mass adherent to the posterior wall, predominantly localized to the left pelvis. Complete resection of the uterus, adnexa, and tumor was achieved (**Figure 1**). Histopathology confirmed uterine leiomyosarcoma, with immunohistochemistry showing SMA (+), Desmin (+), Ki67 (+30%), and negative MDM2, p53, BRCA1, and PD-L1. Adjuvant chemotherapy (six cycles) and genetic testing were advised; however, the patient declined further treatment (including radiotherapy, immunotherapy, targeted therapy) due to socioeconomic constraints. Ureteral stents were removed two months postoperatively.

The patient did not follow clinical follow-up recommendations. Four years after initial resection, she presented with 3-day absence of flatus and bowel movements. Imaging showed a 20-cm pelviperitoneal solid mass, indicative of colonic metastasis, and secondary cytoreductive surgery was performed. After obtaining informed consent for tumor microenvironment studies, scRNA-seq was done on pelvic and colon metastatic lesions. She declined postoperative adjuvant therapy and was discharged after recovery. Nine months after the second surgery, she was readmitted for severe abdominal pain. Pelvic MRI revealed a neoplastic lesion compressing the bladder and rectum; third cytoreduction was performed, showing transmural tumor invasion of the bladder mucosa and peritoneum. ScRNA-seq profiling was conducted on specimens from bladder metastasis and abdominal wall metastasis.

### 3.2 Single-cell RNA sequencing characterized ULSA-related cellular composition within tumor tissues

**Figure 2:**
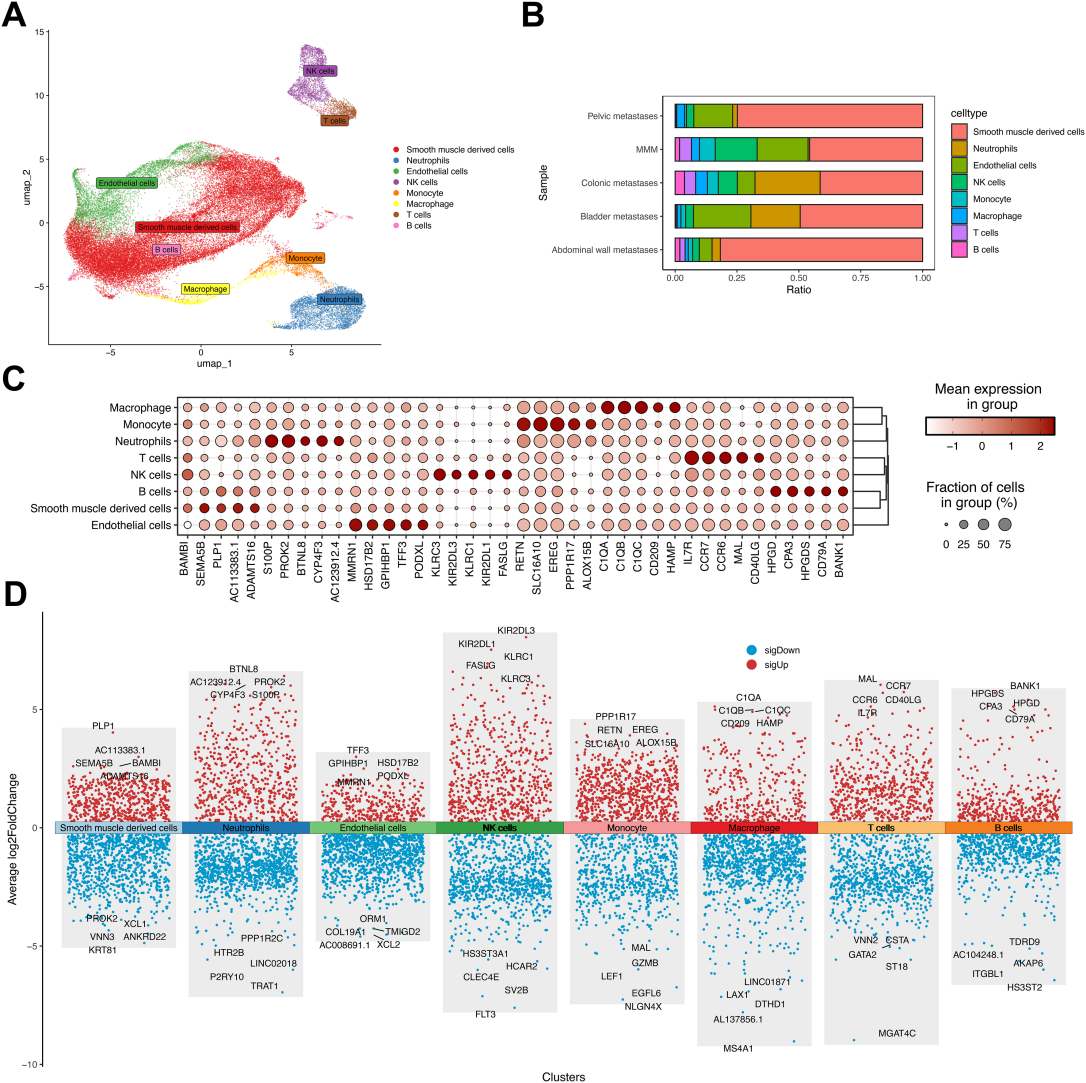
Dimensionality reduction analysis in uterine leiomyosarcoma single-cell profiling. **(A)** UMAP visualization of four metastatic ULSA specimens versus five normal myometrial controls. **(B)** Cellular composition distribution among eight annotated populations across metastatic and control cohorts. **(C)** Top five discriminatory markers per cell lineage. **(D)** Differential gene expression volcano plot highlighting most significantly dysregulated transcripts per population.

To characterize transcriptional alterations in uterine leiomyosarcoma (ULSA), patient-derived samples underwent single-cell RNA sequencing. Metastatic foci from pelvic, rectal, peritoneal, and vesical sites were enzymatically dissociated into single-cell suspensions. Control specimens comprised uterine smooth muscle tissue from five disease-free individuals. The 10× Genomics platform generated 50,818 high-quality transcriptomes post-quality control. Processing involved: low-quality read filtration, CellRanger-based reference genome alignment, gene annotation, and unique molecular identifier (UMI) correction. Uniform manifold approximation and projection (UMAP) visualization resolved eight principal cellular populations (**Figure 2A**), which were annotated using canonical markers including T lymphocytes (CD247/CD3D/CD3E), NK cells (NKG7/PRF1/GZMB), B lymphocytes (MS4A1/IGHM), neutrophils (S100A8/TREM1), endothelial cells (CLDN5), monocytes (CD14/ITGAX), macrophages (CD68/FCGR3A), and smooth muscle derivatives (ACTA2/CALD1/TAGLN) (31). Cellular distribution heterogeneity across samples is depicted in **Figure 2B**. Metastatic cohorts exhibited neutrophilic expansion relative to MMM, particularly prominent in colonic and vesical metastases. This implicates neutrophil-mediated mechanisms in uterine sarcoma dissemination. Abdominal wall metastases demonstrated >80% smooth muscle derivatives, correlating with hematoxylin-eosin staining (**Figure 1**). Lineage-specific top markers include (**Figure 2C**): Macrophage: C1QA; Monocyte: RETN; Neutrophil: S100P; T cell: IL7R; NK cell: KLRC3; B cell: HPGD; Smooth muscle: BAMBI; Endothelial: MMRN1. Volcano plot visualization in **Figure 2D** exhibits significantly dysregulated genes across all eight populations.

### 3.3 Identification of an invasive ULSA cellular subpopulation

**Figure 3:**
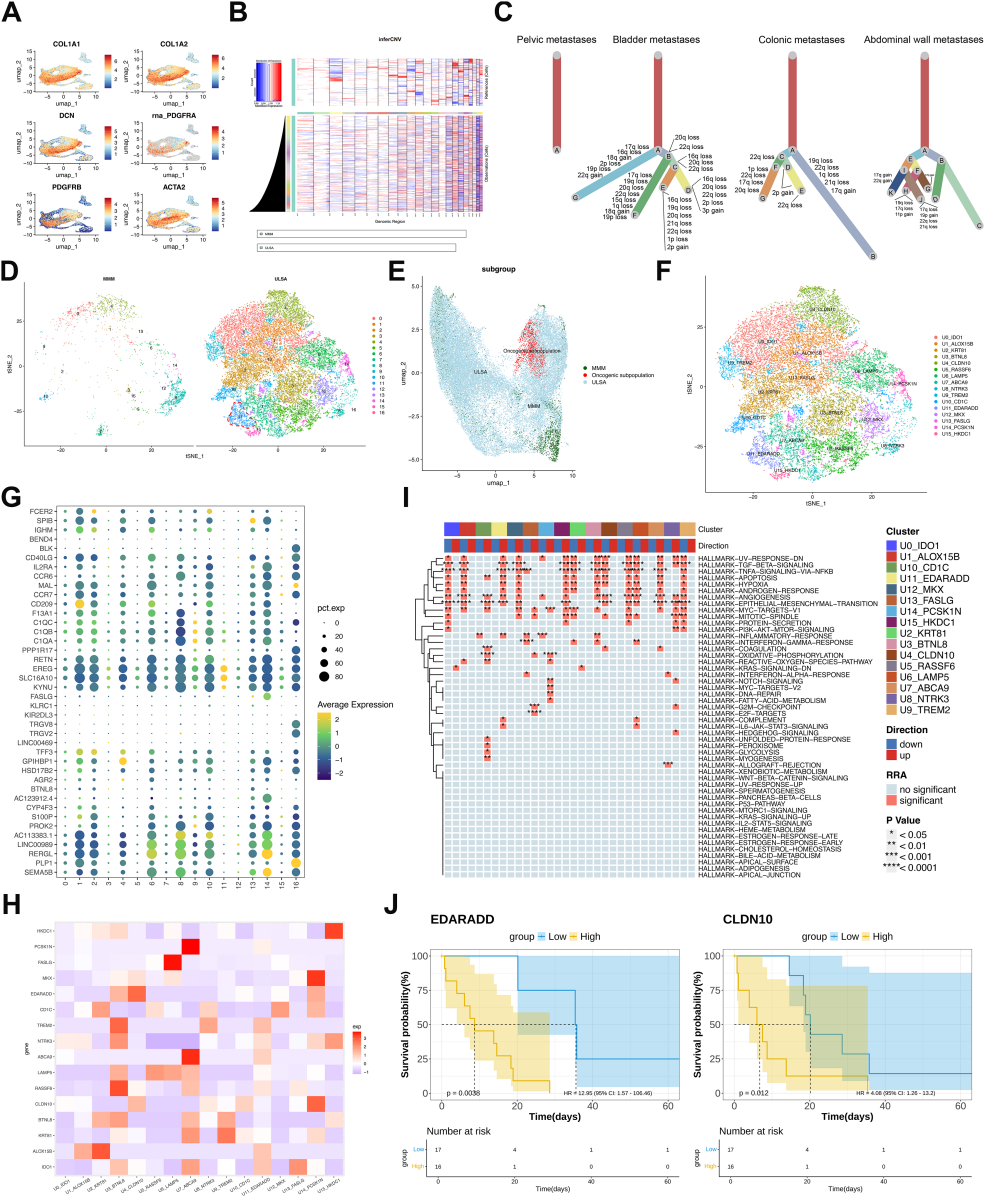
Malignant Cell Characterization and Functional Enrichment in ULSA. **(A)** UMAP visualization of ULSA-defining markers (COL1A1, COL1A2, DCN, PDGFRA, PDGFRB, ACTA2). **(B)** Copy number variation heatmap from inferCNV analysis (red: gain; blue: loss). **(C)** Phylogenetic reconstruction across four metastatic lesions. **(D)** tSNE projection contrasting ULSA and myometrial tissues; dashed circle demarcates metastasis-associated oncogenic subset. **(E)** UMAP representation of oncogenic subpopulation. **(F)** tSNE resolution of 16 cellular subclusters (excluded underpopulated cluster). **(G)** Expression bubble plot across 17 cellular clusters. **(H)** Marker correlation heatmap for 16 subclusters. **(I)** Robust rank aggregation (RRA) enrichment heatmap. **(J)** TCGA-SARC survival stratification by EDARADD/CLDN10 expression (high vs low groups).

**Figure 4:**
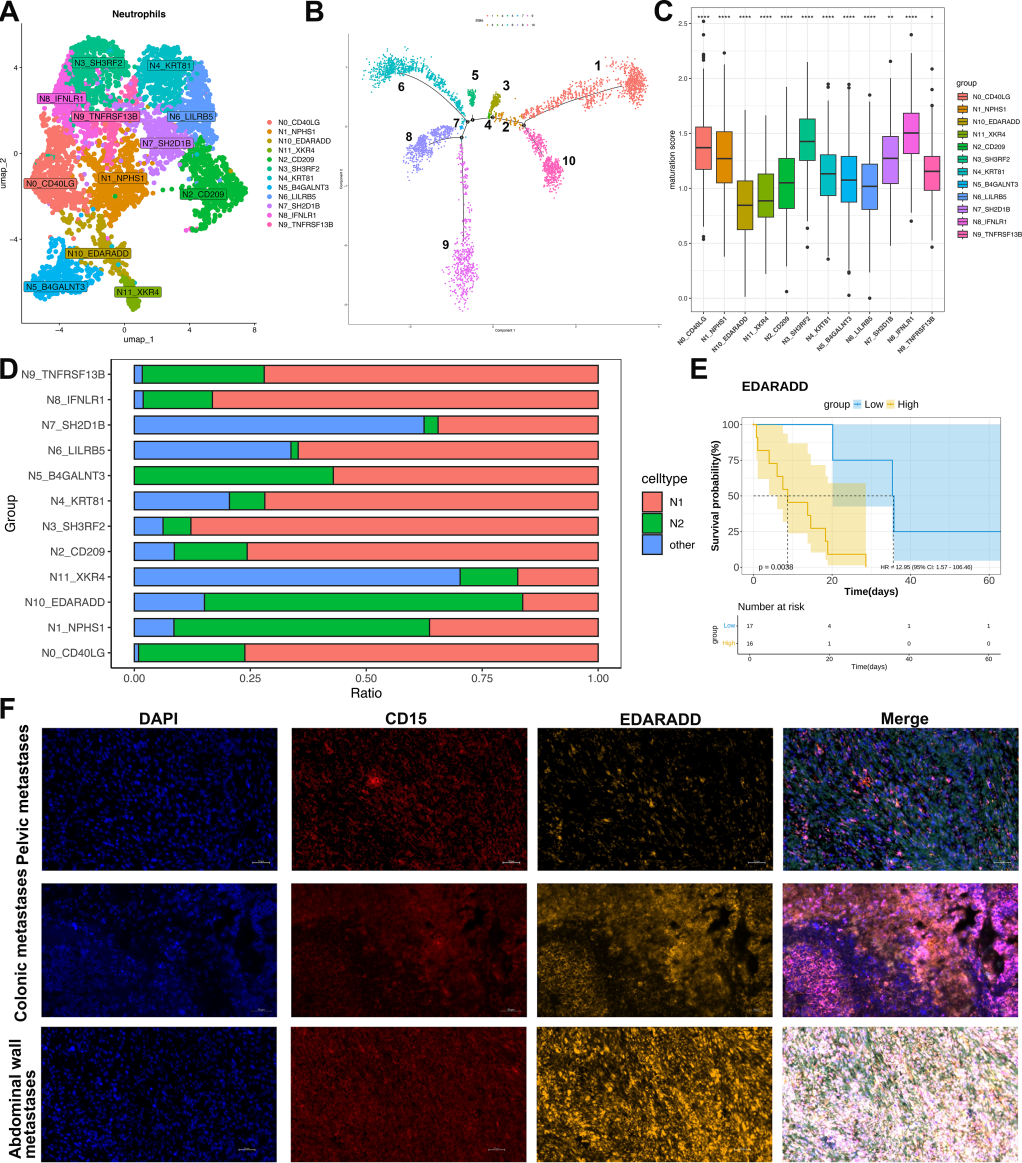
Characterization and pseudotemporal ordering of neutrophil subsets in ULSA. **(A)** Neutrophil subpopulation classification within ULSA defined twelve distinct clusters: N0_CD40LG, N1_NPHS1, N2_CD209, N3_SH3RF2, N4_KRT81, N5_B4GALNT3, N6_LILRB5, N7_SH2D1B, N8_IFNLR1, N9_TNFRSF13B, N10_EDARADD, N11_XKR4. **(B)** Pseudotemporal reconstruction resolved neutrophil developmental trajectories across ten discrete states. **(C)** Maturation scoring applied to neutrophil subpopulations. **(D)** Relative abundance of N1-polarized versus N2-polarized neutrophils among identified subsets. **(E)** Patient survival stratified according to median expression levels of neutrophil subcluster-defining markers. **(F)** The mpIF validating CD15^+^EDARADD^+^ neutrophil immunolocalization in pelvic, colonic, and abdominal wall metastatic lesions. *P<0.05, **P<0.01, ****P<0.0001.

Malignant cells were distinguished from normal counterparts within smooth muscle derivatives using ULSA markers (32)(COL1A1, COL1A2, ACTA2, PDGFRA, PDGFRB, DCN; **Figure 3A**) alongside inferCNV-based copy number variation assessment (**Figure 3B**). Tumor phylogenetics demonstrated multi-branch evolution in bladder, colonic, and abdominal wall metastases (**Figure 3C**), exhibiting recurrent chromosomal alterations across lineages. Contrastingly, pelvic wall metastases displayed singular evolutionary trajectories, aligning with recurrence chronology and implantation metastasis sequences. tSNE analysis resolved 17 cellular subpopulations across ULSA and myometrial tissues, refined to 16 clusters following exclusion of one underpopulated subset (**Figure 3D**), highlighting the inherent heterogeneity of ULSA. Notably, cluster 11 (1,182 cells) appeared exclusively in ULSA specimens (**Figures 3D, E**), concentrated primarily within abdominal wall metastases, suggesting its involvement in distant dissemination. Cluster 11 (U11) exhibited EDARADD expression (**Figure 3F**), a TNFR superfamily member implicated in metastatic progression and adverse prognosis across malignancies. U11-EDARADD concurrently demonstrated elevated EREG and SLC16A10 transcription (**Figure 3G**), both recognized oncogenic drivers. Correlation heatmap revealed that EDARADD expression was correlated with U4-CLDN10 (**Figure 3H**). Functionally, tumor-promotive pathways including TGF-β signaling, angiogenesis, epithelial-mesenchymal transition, and TNF-α/NF-κB activation were significantly enriched in cluster 11 (**Figure 3I**). TCGA-SARC survival analysis incorporating cluster-specific gene signatures established that elevated EDARADD and CLDN10 expression correlated with diminished survival (*P*<0.05, **Figure 3J**), validating prior observations. To validate these findings, co-staining analysis of EDARADD and CLDN10 in previous specimens showed similar and consistent results (**Figure 4F**, **Supplementary Figure S1**). Overall, the U11-EDARADD cell subset exhibits certain invasive characteristics, with enhanced metastatic ability and potentially significant prognostic implications.

### 3.4 Neutrophil compartmental diversity during developmental trajectories

To elucidate neutrophil heterogeneity, we collected 4,535 high-quality neutrophils post-quality control and classified them into twelve distinct lineages (N0-N11, **Figure 4A**). Pseudotemporal ordering via Monocle revealed a tightly orchestrated differentiation trajectory. This trajectory initiated from the N10-EDARADD, N11-XKR4, and N8-IFNLR1 branches and terminated at lineages N3-SH3RF2, N4-KRT81, N6-LILRB5, and N7-SH2D1B (**Figure 4B**). Neutrophil maturation scores were computed using established differentiation-associated genes (37)(**Figure 4C**). Lineages positioned at the trajectory origin (N10-EDARADD and N11-XKR4) exhibited minimal maturation scores. Intermediate scores characterized N2-CD209, N6-LILRB5, N4-KRT81, and N5-B4GALNT3, whereas maximal scores defined N8-IFNLR1 and N3-SH3RF2. Functional polarization was evaluated using N1/N2-associated markers (38)(**Figure 4D**). The highly mature N8-IFNLR1 and N3-SH3RF2 lineages predominantly displayed an N1 phenotype (~90% N1 neutrophils). Conversely, the minimally mature N10-EDARADD lineage demonstrated N2 polarization (>70% N2 neutrophils). Substantial proportions of non-polarizable neutrophils populated the N7-SH2D1B and N11-XKR4 lineages.

Survival analysis incorporating lineage-specific markers (**Figure 4E**) indicated that elevated expression of the N10 marker EDARADD correlated with adverse patient outcomes (*P*<0.05), implicating pro-tumor effects from immature, N2-polarized neutrophils in ULSA. Validation employed mpIF on metastatic tissue sections (pelvic, colon, abdominal wall metastases) (**Figure 4F**). CD15^+^EDARADD^+^cells (orange: CD15 [red], EDARADD [yellow]) appeared in all samples. These round or oval cells localized within tumor stroma, adjacent to carcinoma or stromal cells. Notably, colon and abdominal wall metastases exhibited significantly enhanced CD15^+^EDARADD^+^cell density and fluorescence intensity versus pelvic metastases, with abdominal wall lesions demonstrating widespread positivity and cell cluster formation.

### 3.5 Angiogenic and metastatic propensity in malignant endothelial subsets

**Figure 5:**
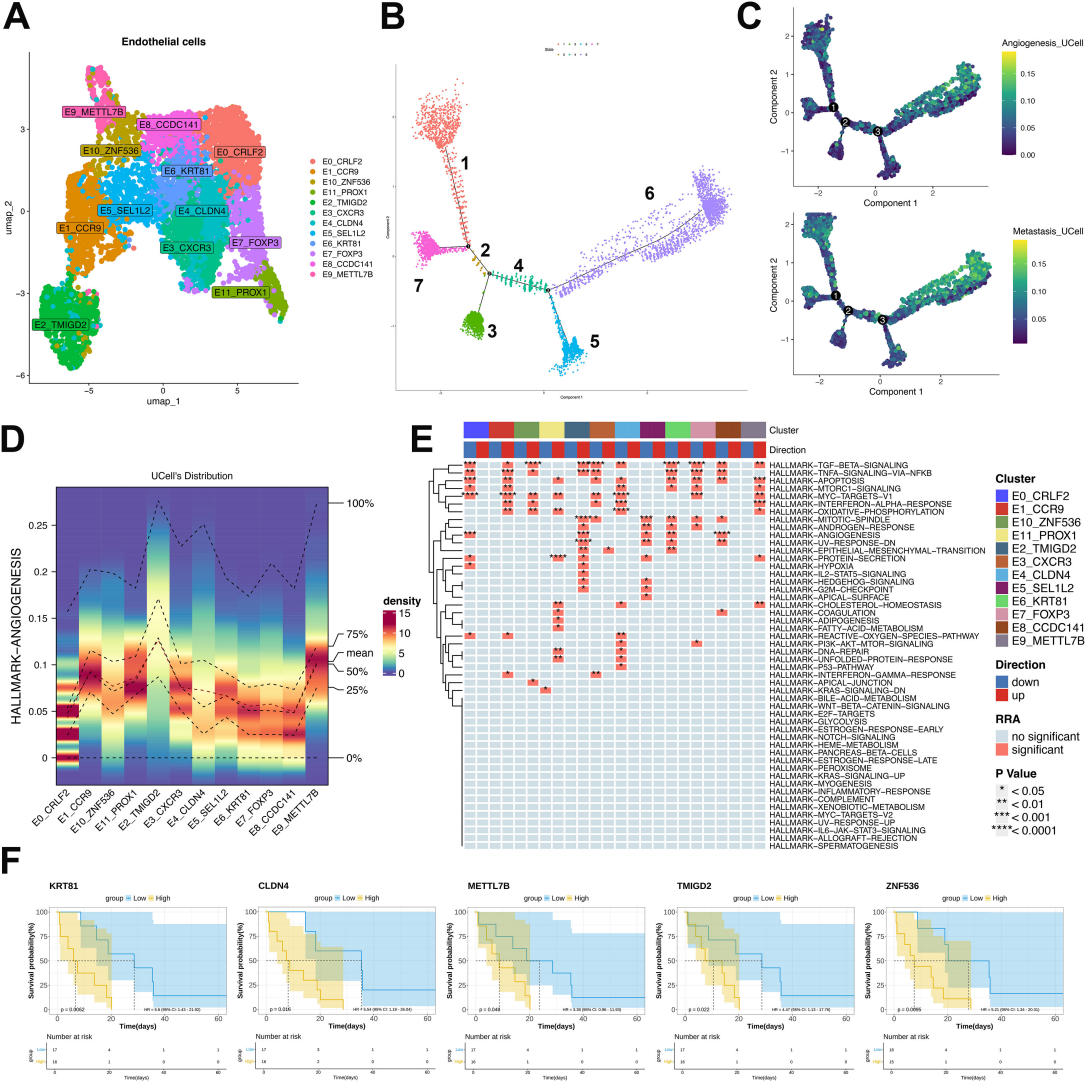
Endothelial subpopulation characterization and pseudotemporal dynamics in ULSA. **(A)** Twelve malignant endothelial clusters resolved via clustering analysis. **(B)** Seven cellular states derived from pseudotemporal ordering. **(C)** Angiogenic and metastatic trajectory dynamics in endothelial monocle analysis. **(D)** Heatmap evaluating expression patterns and consistency of angiogenesis-associated genes across twelve subtypes. **(E)** Robust rank aggregation (RRA) enrichment heatmap. **(F)** TCGA-SARC survival analysis of malignancy-associated transcripts.

Pseudotemporal trajectory analysis resolved twelve malignant endothelial clusters (**Figure 5A**) and seven cellular states (**Figure 5B**). Cluster-defining markers revealed distinct state distributions: E3-CXCR3, E6-KRT81, E7-FOXP3, E8-CCDC141 and E0-CRLF2 predominated in early states, whereas E1-CCR9, E10-ZNF536 and E11-PROX1 accumulated in terminal states. E2-TMIGD2 and E9-METTL7B strongly associated with angiogenic and metastatic potential (**Figures 5C–E**). E4-CLDN4 and E5-SEL1L2 exhibited pan-state distribution without bias (**Figure 5C**). Functional assessment identified TGF-β, TNF/NF-κB angiogenesis and EMT pathways as significantly enriched in terminal-state clusters (E1, E2, E9-E11) (**Figure 5E**). Survival analysis of malignancy-associated transcripts (**Figure 5F**) within the TCGA-SARC ULSA cohort demonstrated significantly reduced overall survival in patients exhibiting elevated expression of endothelial cluster genes (TMIGD2, KRT81, CLDN4, METTL7B, ZNF536; *P*<0.05). This correlation suggests that endothelial enrichment in latent states, 5, 6, and 7, is likely to predict poor clinical outcomes.

### 3.6 The analysis of T and B cell subsets revealed an immunosuppressive tumor microenvironment in ULSA patients

**Figure 6:**
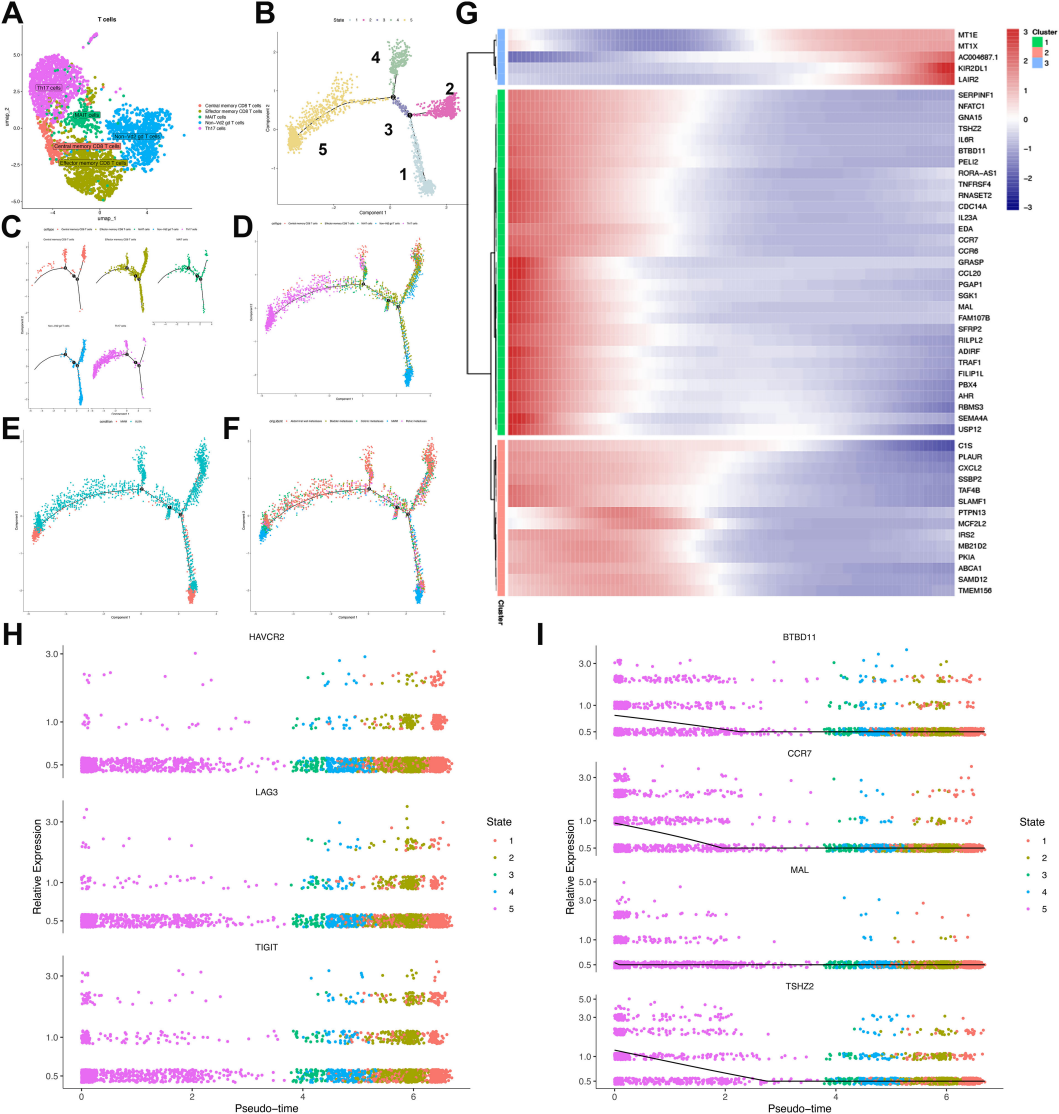
Characterization and pseudotemporal ordering of T lymphocyte subpopulations in ULSA. **(A)** Unsupervised clustering delineated five principal T-cell subsets: MAIT cells, effector memory. CD8^+^ T cells, central memory CD8^+^ T cells, Th17 cells, and non-Vδ2 γδ T cells. **(B)** Cellular developmental trajectories resolved through pseudotime analysis revealed five discrete states. **(C)** Monocle-derived trajectory visualization stratified by cellular phenotypes. **(D)** Pseudotemporal progression mapping organized according to immunophenotypes. **(E)** Cell trajectory arrangement classified through subgroup partitioning. **(F)** Developmental pathway representation segregated by sample cohorts. **(G)** Heatmap delineating expression patterns of 50 most significant differentially expressed genes (lowest q-values). **(H)** Pseudotime-dependent expression profiles of exhaustion markers (HAVCR2, LAG3, TIGIT) across identified states. **(I)** Relative abundance of trajectory-associated gene markers.

To elucidate intrinsic cellular organization and potential functional states within T-cell populations, we performed unsupervised clustering using UMAP visualization. Five distinct clusters were identified, encompassing effector memory CD8^+^ T cells, central memory CD8^+^ T cells, mucosal-associated invariant T (MAIT) cells, Th17 cells, and non-Vδ2 γδ T cells (**Figure 6A**). Developmental trajectories of T cells in ULSA were reconstructed using reverse graph embedding to position cells along a pseudotemporal continuum. Pseudotime ordering delineated five cellular states, with Th17 and central memory CD8^+^ T cells occupying initial pseudotime positions. Effector memory CD8^+^ T cells and MAIT cells populated subsequent developmental stages, while non-Vδ2 γδ T cells emerged as discrete lineages at states 4 and 5 (**Figures 6B–F**). Spatial mapping demonstrated MMM cell accumulation in regions enriched for Th17 and non-Vδ2 γδ T cells. Conversely, ULSA cells predominantly colocalized with central memory CD8^+^ T cells, effector memory CD8^+^ T cells, and MAIT cell populations (**Figures 6D, E**). Longitudinal profiling revealed progressive depletion of naïve T cells defined by TSHZ2, CCR7, MAL and BDBD11expression along the pseudotemporal trajectory (**Figures 6G, I** **).** Conversely, terminally exhausted CD8^+^ T cells expressing canonical exhaustion markers LAG3, HAVCR2 and TIGIT demonstrated substantial enrichment during later pseudotime intervals, indicating a phenotypic transition from activated to exhausted states in CD8^+^ T lymphocytes (**Figures 6G, H**). Pseudotemporal analysis identified three distinct transcriptional clusters among the top 50 differentially expressed genes (DEGs) in CD8^+^ T cell trajectories (**Figure 6G**). Cluster 1 exhibited ascending expression of metallothioneins (MT1X, MT1E), KIR2DL1, and the exhaustion biomarker LAIR2 toward trajectory endpoints. Conversely, naïve T-cell markers (CCR7, MAL, BDBD11) within Cluster 2 displayed progressive downregulation. Cluster 3 contained decreasing regulators including CXCL2, PLAUR, and TAF4B. The observed enrichment of exhausted T cells coupled with declining naïve T-cell frequencies and diminished regulatory molecule expression along developmental trajectories collectively indicates establishment of an immunosuppressive microenvironment during tumor progression and metastatic dissemination.

**Figure 7:**
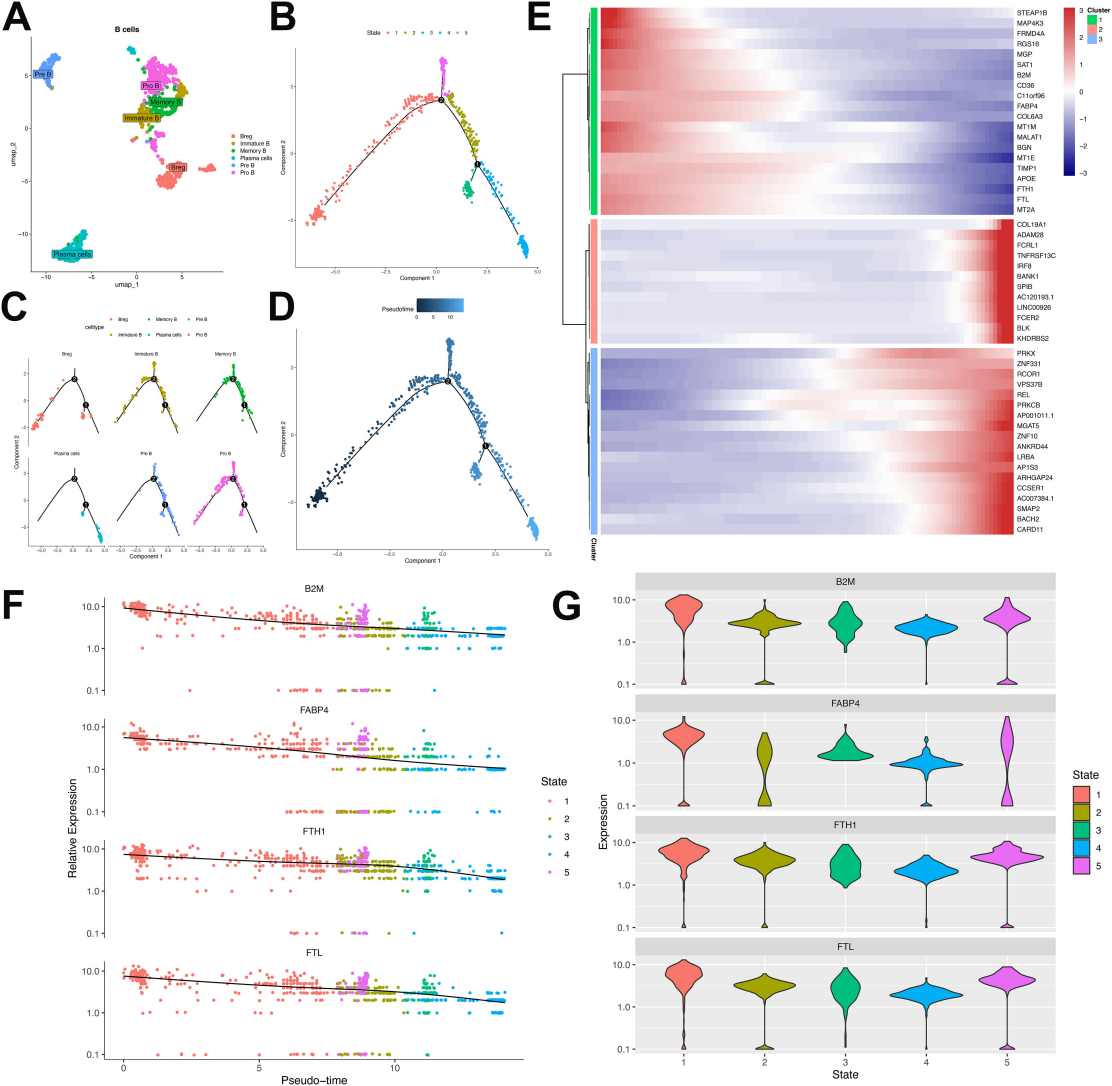
Characterization and developmental ordering of B lymphocyte subpopulations in ULSA. **(A)** Unsupervised clustering resolved six principal B-cell subsets: plasma cells, immature B cells, pre-B cells, pro-B cells, memory B cells, and regulatory B cells (Breg). **(B)** Pseudotemporal ordering classified B lymphocytes into five discrete developmental states. **(C)** Monocle-derived trajectory representing developmental progression of the six B-cell subsets. **(D)** Evolutionary pathways of B lymphocytes documented through pseudotime analysis. **(E)** Heatmap illustrating expression patterns of 50 most significant differentially expressed genes (lowest q-values). **(F)** Expression dynamics of B2M, FABP4, FTH1, and FTL throughout pseudotemporal states. **(G)** Violin plots depicting expression distributions of B2M, FABP4, FTH1, and FTL across developmental states.

Comprehensive B-cell profiling of 797 cells identified six distinct lineages—Breg, plasma B, pre B, pro B, memory B, and immature B cells—within this patient’s tumor microenvironment (**Figure 7A**). Substantial inter-sample heterogeneity in lineage distribution (**Supplementary Figure S2**) suggested varied humoral immunity across metastatic lesions. Developmental trajectory reconstruction via pseudotime ordering revealed progressive maturation from initial Pro-B/Breg branches toward immature B, pre B, memory B, and plasma cell states (**Figures 7B–D**).

Trajectory analysis of B lymphocytes resolved three clusters comprising the top 50 differentially expressed genes (DEGs) (**Figure 7E**). Cluster 1, defined by markers associated with B-cell proliferation and development (B2M, FTH1, FABP4, FTL) (33), exhibited progressive diminution across the trajectory (**Figures 7E–G**). B2M constitutes an essential component of major histocompatibility complex class I (MHC-I), critical for antigen presentation. Tumor-infiltrating B cells may downregulate B2M to evade MHC-I-mediated immune surveillance. FABP4 modulates B-cell functionality within tumor microenvironments and potentially mediates crosstalk with immunosuppressive populations (e.g., tumor-associated macrophages, regulatory T-cells), fostering tumor-permissive niches. Ferritin components FTH1 and FTL contribute to malignant B-cell persistence by regulating iron storage, thereby supporting heightened metabolic demands during neoplastic proliferation. Cluster 2 demonstrated progressive enrichment, encompassing cells expressing canonical B-cell markers (Bank1, BLK), memory markers (FCRL1), and regulatory factors IRF8/SPIB that direct progenitor differentiation. Elevated expression of FCER2 (enhancing antibody production) and KHDRBS2 (ensuring functional BCR development) further characterized this cluster. Cluster 3 featured transcriptional regulators (PRKX, ZNF10, PRKCB, ZNF331, RCOR1) governing B-cell maturation and antibody synthesis, accumulating at trajectory termini. Cell-cell interaction analysis implicated the MIF-(CD74+CD44) ligand-receptor was involved in T-cell and B-cell crosstalk (**Supplementary Figure S3**). Tumor cells potentially exploit MIF-(CD74+CD44) signaling to subvert immune surveillance, wherein MIF binding impairs immune effector functions. Collectively, these findings delineate B-cell heterogeneity in antitumor immunity and underscore the immunosuppressive landscape of ULSA.

### 3.7 Compartmental heterogeneity among myeloid cell lineages in ULSA

**Figure 8:**
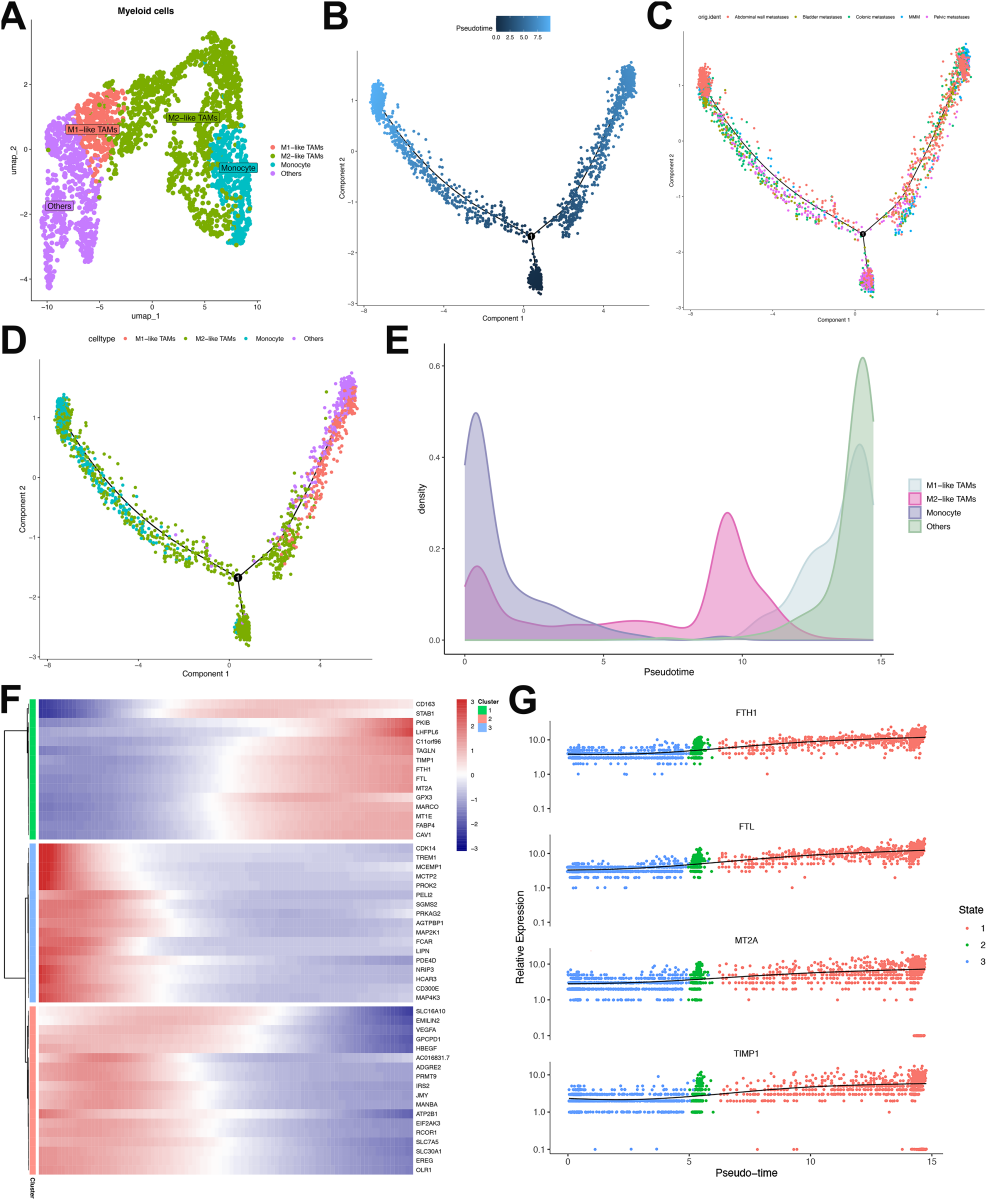
Characterization and developmental ordering of myeloid cell subpopulations in ULSA. **(A)** The clusters were identified as 4 myeloid cell subgroups: Monocytes, M1-like TAMs, M2-like TAMs, and Others. **(B)** Pseudotemporal ordering of myeloid cell developmental progression. **(C)** Monocle-derived trajectory visualization stratified by sample cohorts. **(D)** Evolutionary pathways representing seven distinct myeloid subpopulations. **(E)** Pseudotime-dependent cellular density distribution mapping. **(F)** Heatmap illustrating expression patterns of 50 most significant differentially expressed genes (lowest q-values). **(G)** Expression dynamics of FTH1, FTL, MT2A, and TIMP1 across pseudotemporal states.

Comprehensive profiling of 2,401 myeloid cells resolved four distinct categories, namely: M1-like tumor-associated macrophages (TAMs), M2-like TAMs, monocytes, and others (**Figure 8A**). Pseudotemporal trajectory analysis segregated these cells into three discrete developmental states, revealing differential distribution patterns across cell types and sample origins (**Figures 8B–D**). Trajectory reconstruction indicated monocyte differentiation into M1-like TAMs and M2-like TAMs (**Figure 8E**). Transcriptional clustering of the top 50 trajectory-associated DEGs identified three signature groups (**Figure 8F**). Cluster 1 exhibited progressive enrichment of CD163 (a canonical TAM/M2 marker) alongside TAM-associated genes FTH1, FTL, MT2A, and TIMP1 (34) (**Figures 8F, G**). Conversely, Cluster 2 demonstrated declining expression of M1-polarization regulators TREM1, PDE4D, MCEMP1 and MAP4D (35, 36), indicating directional polarization shift from M1 to M2 phenotypes in ULSA (**Figure 8F**). Cluster 3 displayed terminal diminution of M2-associated markers (VEGFA, HBEGF, ADGRE2, EMILIN2) along the trajectory, suggesting the coexistence of M1 and M2 macrophages in myeloid cells in ULSA.

Cellular interaction analysis implicated CXCL8-ACKR1 signaling in monocyte-endothelial cross-talk (**Supplementary Figure S4**), where aberrant ACKR1 expression elevates CXCL8 to promote angiogenesis, metastasis, and TAM polarization toward immunosuppressive M2 states. Neutrophil-monocyte interactions involved ICAM1-(ITGAV+ITGB2) pairing (**Supplementary Figure S4**), with dysregulation promoting pro-tumor TAM differentiation. THBS pathway activation mediated monocyte-smooth muscle communication (**Supplementary Figure S4**), driving pro-tumorigenic M2-like polarization to facilitate tumor growth. Collectively, these data demonstrate myeloid compartment heterogeneity in ULSA while highlighting convergent pro-tumorigenic functions across distinct lineages.

## 4 Discussion

ULSA is associated with a poor prognosis, demonstrating high rates of local and distant recurrence and a median overall survival of only two years following metastasis (39). Current treatment modalities, including surgical resection combined with chemotherapy and radiotherapy (40), as well as immunotherapies such as immune checkpoint blockade, show limited efficacy in ULSA patients (10, 41). The development of more effective therapies has been hindered by inadequate understanding of tumor heterogeneity and the complex immune microenvironment (42, 43). To address this, analyzed scRNA-seq data from a ULSA patient and five non-tumor patients, generating a comprehensive profile of ULSA and MMM microenvironments at single-cell resolution. Our findings identify CD8^+^ T cell exhaustion, pro-tumor M2 macrophages, and N2-polarized neutrophils as key contributors to the immunosuppressive ULSA microenvironment.

A distinct U11 subpopulation in ULSA may play a pivotal role in metastatic dissemination. Single-cell analysis revealed tumor heterogeneity during ULSA progression, identifying subclusters with divergent functional properties. Among these, the U11-EDARADD cluster, enriched in EMT and angiogenesis signatures, was predominantly localized in abdominal wall metastases. EMT activation drives tumor invasion, dissemination, and therapy resistance, contributing to aggressive disease progression (43–45). Furthermore, U11 exhibited marked upregulation of TNF-α-induced NF-κB signaling, a pathway known to enhance cancer cell invasiveness and metastatic potential (46–48). Prior studies in melanoma demonstrate that the TNF-α/NF-κB/MMP9 axis promotes early metastasis by facilitating detachment from primary tumors and systemic dissemination via vascular or lymphatic routes (49–51). Clinically, these mechanisms align with the observed rapid disease course in the patient, who developed two recurrences with multiorgan metastases within 17 months, reflecting a highly aggressive phenotype.

ULSA exhibits a profoundly immunosuppressive tumor microenvironment. Our results demonstrate a phenotypic shift in CD8^+^ T cells from activated to exhausted states, a process driven by chronic antigenic and inflammatory stimulation during tumor progression. Exhausted CD8^+^ T cells are characterized by co-expression of inhibitory receptors (HAVCR2, LAG3, TIGIT) (52), diminished memory recall, and progressive functional decline in cytokine secretion and cytotoxic activity, collectively promoting immune evasion (53, 54). Despite therapeutic efforts to reverse T-cell exhaustion, sustained recovery remains elusive, with frequent relapse to exhausted states post-treatment (55). Although immune checkpoint inhibitors (anti-PD1/anti-CTLA4) are foundational in immunotherapy, they often fail to achieve durable responses in ULSA (39). Ligand-receptor interaction analysis identified MIF-(CD74+CD44) as a key mediator of T-cell–B-cell crosstalk. MIF acts as a pleiotropic immunomodulator, influencing both inflammatory and oncogenic pathways (56, 57). Upon binding to (CD74+CD44) complexes, extracellular MIF triggers downstream signaling that exacerbates inflammation, tumor growth, and metastasis (58). Intracellularly, MIF further regulates Toll-like receptor signaling and inflammatory cascades (59). Our findings implicate MIF-(CD74+CD44) axis activation as a mechanistic contributor to ULSA pathogenesis.

Macrophage activation polarizes cells into M1-like (pro-inflammatory) and M2-like (immunoregulatory) phenotypes, both critical mediators of inflammatory responses (60). In tumor microenvironments, tumor-associated macrophages (TAMs) primarily exhibit an M2-like phenotype, driving immunosuppression and metastatic progression through upregulated signaling pathways (61, 62). Our findings demonstrate coexisting M1-like and M2-like macrophage populations in ULSA, with M2-like subsets dominating the myeloid infiltrate. The observed M1-to-M2 shift implies that M2-like TAMs play a pivotal role in ULSA pathogenesis and immune escape. Importantly, we discovered that ICAM1 binding to the ITGAX/ITGB2 heterodimer facilitates neutrophil-monocyte crosstalk, skewing monocyte differentiation toward pro-tumor TAMs and fostering tumorigenesis (63, 64). Consequently, targeting ITGAX/ITGB2-ICAM1 interactions may represent a viable strategy to block immunosuppressive TAM recruitment and function (65).

Neutrophils demonstrate functional duality within tumor microenvironments, a phenomenon long overlooked due to technical limitations in isolation and phenotypic characterization (66, 67). Recent advances leverage maturation indices and N1/N2 polarization frameworks to resolve their functional heterogeneity (68, 69). In ULSA, immature neutrophils with low maturation scores predominantly adopt N2 polarization, whereas mature subsets retain N1 phenotypes—consistent with evidence linking tumor-infiltrating immature neutrophils to pro-oncogenic functions (70). This dichotomy corroborates the established N1 (antitumor) and N2 (protumor) paradigm (71), illustrating their plasticity in adapting to microenvironmental cues to either suppress or promote tumor progression (72).

Under the influence of TGF-β, IL-8, IL-6, and IL-17, neutrophils polarize into the N2 subtype, characterized by prolonged lifespan, an immature phenotype, reduced cytotoxicity, and pro-tumor functions, including promotion of tumor growth, invasion, metastasis, angiogenesis, and immune suppression (73). N2 neutrophils exhibit pro-tumor activity, primarily through the secretion of arginase, matrix metalloproteinase-9 (MMP-9), vascular endothelial growth factor (VEGF), and various chemokines, which facilitate tumor metastasis and angiogenesis within the tumor microenvironment (74, 75). Retrospective studies have identified significant differences in absolute neutrophil count (ANC) and neutrophil-to-lymphocyte ratio (NLR) between leiomyoma and leiomyosarcoma, with elevated NLR strongly correlating with poor overall survival in sarcomatoid analyses (76, 77). CD74, a key receptor for macrophage migration inhibitory factor (MIF), has been implicated in cancer prognosis; CD74^+^ neutrophils are associated with improved patient outcomes in multiple malignancies by inducing antigen-specific T-cell responses and fostering an immunogenic (“hot”) tumor microenvironment, suggesting their potential as an immunotherapy-sensitizing strategy (78, 79). However, the interplay between N2 neutrophils and the MIF/CD74 regulatory axis remains insufficiently explored.

The mpIF analysis demonstrated significant enrichment of CD15^+^EDARADD^+^ neutrophils in colonic, and abdominal wall metastases, implicating this subset in pro-metastatic processes. CD15, an adhesion molecule predominantly expressed on immature N2 neutrophils, has been shown to actively promote tumorigenic progression (80). EDARADD, a transcriptional co-regulator associated with EGFR signaling pathways, appears to orchestrate multiple metastatic processes including epithelial-mesenchymal transition (EMT), tumor proliferation, invasion, angiogenesis, extravasation, and T-cell suppression through EGFR activation, cytokine production, and extracellular matrix modulation (81–83). Clinical correlation analyses revealed that elevated EDARADD expression in neutrophils significantly associates with poorer patient outcomes, positioning CD15^+^EDARADD^+^ neutrophils as a potential therapeutic target for metastasis suppression. The observed accumulation of these neutrophils at metastatic sites may reflect their role in circulating tumor cell entrapment during premetastatic niche formation, consistent with the distinct metastasis patterns documented in colonic and bladder tissues (84–86). Collectively, neutrophil plasticity dictates functional complexity, with immature subsets potentially driving detrimental outcomes during target therapy.

While this study represents the first application of single-cell sequencing to investigate the genetic heterogeneity and tumor microenvironment across metastatic lesions and tumor cells in uterine leiomyosarcoma (ULSA), several limitations must be acknowledged. First, the analysis included metastatic specimens from only one patient and five myometrial control samples, as the rarity and diagnostic complexity of ULSA constrained the cohort size. Additionally, the scarcity of ULSA cases and the absence of publicly available RNA-sequencing datasets for metastatic survival analysis precluded a comprehensive meta-analysis, necessitating reliance on the TCGA-SARC cohort for exploratory survival assessments. Furthermore, the dynamic changes in immune cell populations within metastases are inherently nonlinear, involving multifaceted processes such as migration, local reprogramming, and microenvironmental crosstalk. Monocle’s linear differentiation model may oversimplify this complexity by artificially representing it as a “pseudo-continuous spectrum,” particularly in samples enriched with mature cells. Future studies should integrate multi-omics validation, microenvironmental signaling analysis, and nonlinear computational approaches to more accurately reconstruct the immune ecology of ULSA. Finally, the functional properties of the identified cell clusters and their associated signaling pathways need to be confirmed by expanding sample size through continuous case collection and experimental validation in the future.

## 5 Conclusion

In our research, this pioneering investigation revealed the inaugural single-cell transcriptomic atlas of ULSA, delineating microenvironmental characteristics and metastasis-associated cellular subpopulations to inform future potential therapeutic targeting. The ULSA microenvironment exhibits marked immunosuppression, evidenced by exhausted CD8^+^ T cell populations, predominant M2 macrophage polarization, and prevalent N2 neutrophil infiltration. This immunophenotypic profile potentially underlies the limited clinical efficacy observed with monotherapeutic immune checkpoint inhibition. Combinatorial approaches integrating multimodal immunotherapy with conventional chemotherapy may represent viable treatment avenues. Our findings elucidate molecular aberrations and tumor microenvironmental dynamics in ULSA, providing foundational insights for advancing precise interventional strategies.

## Data Availability

The raw data supporting the conclusions of this article will be made available by the authors, without undue reservation.

## Supplementary material

Supplementary Figure 1 Immunostaining images of CLDN10 in metastases in the pelvis, colon, and abdominal wall. The scale bar is 100 μm.

Supplementary Figure 2 B cell distribution across pseudotime in primary and four metastatic foci.

Supplementary Figure 3 Intercellular interactions among eight cell types with key receptor-ligand pair identification.

Supplementary Figure 4 Critical signaling pathways identified through cellular communication analysis. **(A)** Significant upregulation of CXCL8-ACKR1 signaling in monocyte-endothelial cell interactions. **(B)** Interaction strength for CXCL2-ACKR1 between monocytes and endothelial cells. **(C)** ICAM signaling network activity across all eight cell types. **(D)** ICAM1-(ITGAX+ITGB2) mediated communication within the cellular cohort. **(E)** THBS signaling pathway network among the characterized cell populations.
