# Supplementary figures and images for: Single-cell profiling delineates the tumor microenvironment and immunological networks in patient-derived uterine leiomyosarcoma
Source: Front Immunol. 2025 Aug 29;16:1653096. doi: 10.3389/fimmu.2025.1653096 (PMC12426000; doi:10.3389/fimmu.2025.1653096)

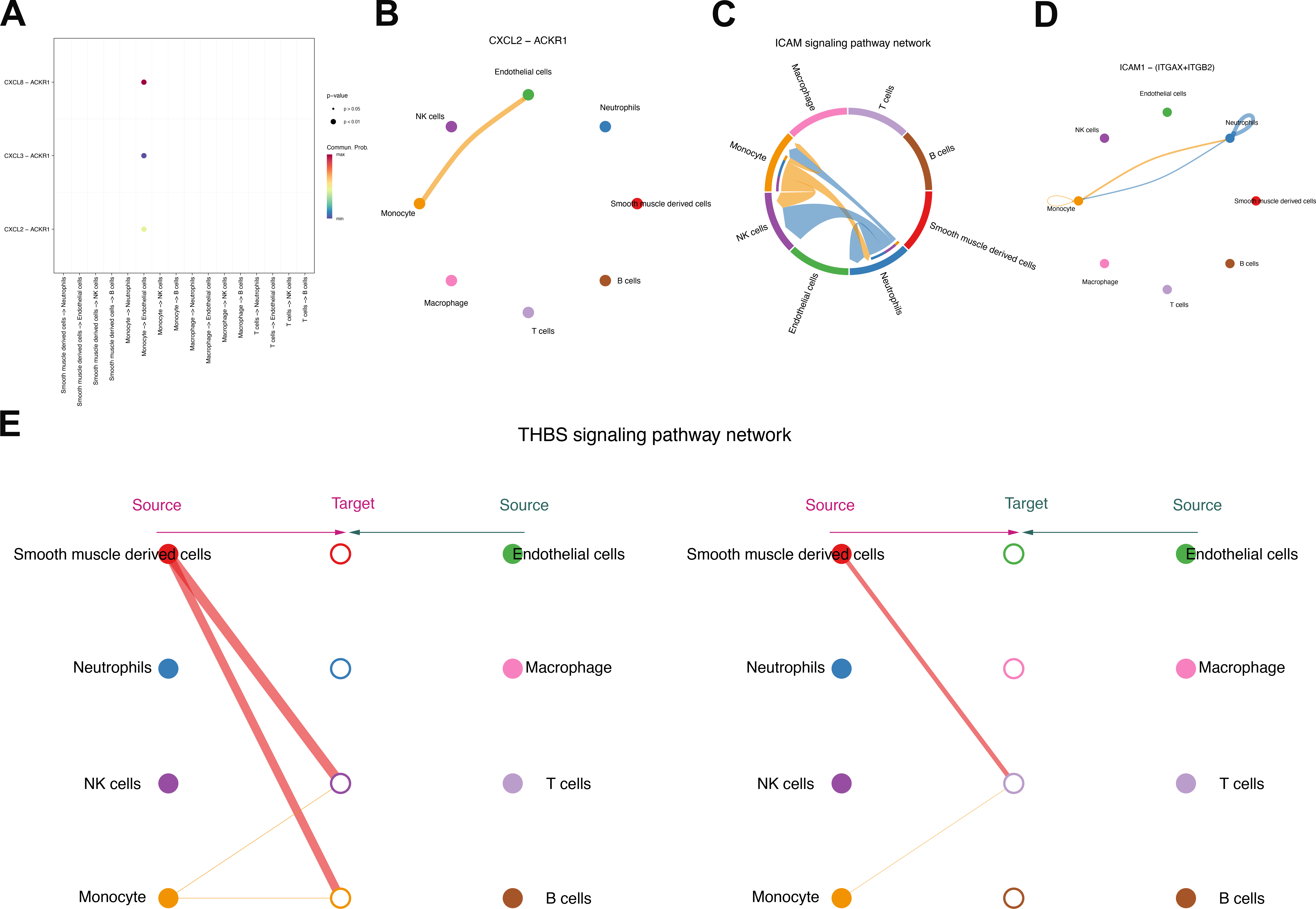

Supplement: Supplementary Figure 1 — Immunostaining images of CLDN10 in metastases in the pelvis, colon, and abdominal wall. The scale bar is 100 μm. [file Image1.tif]

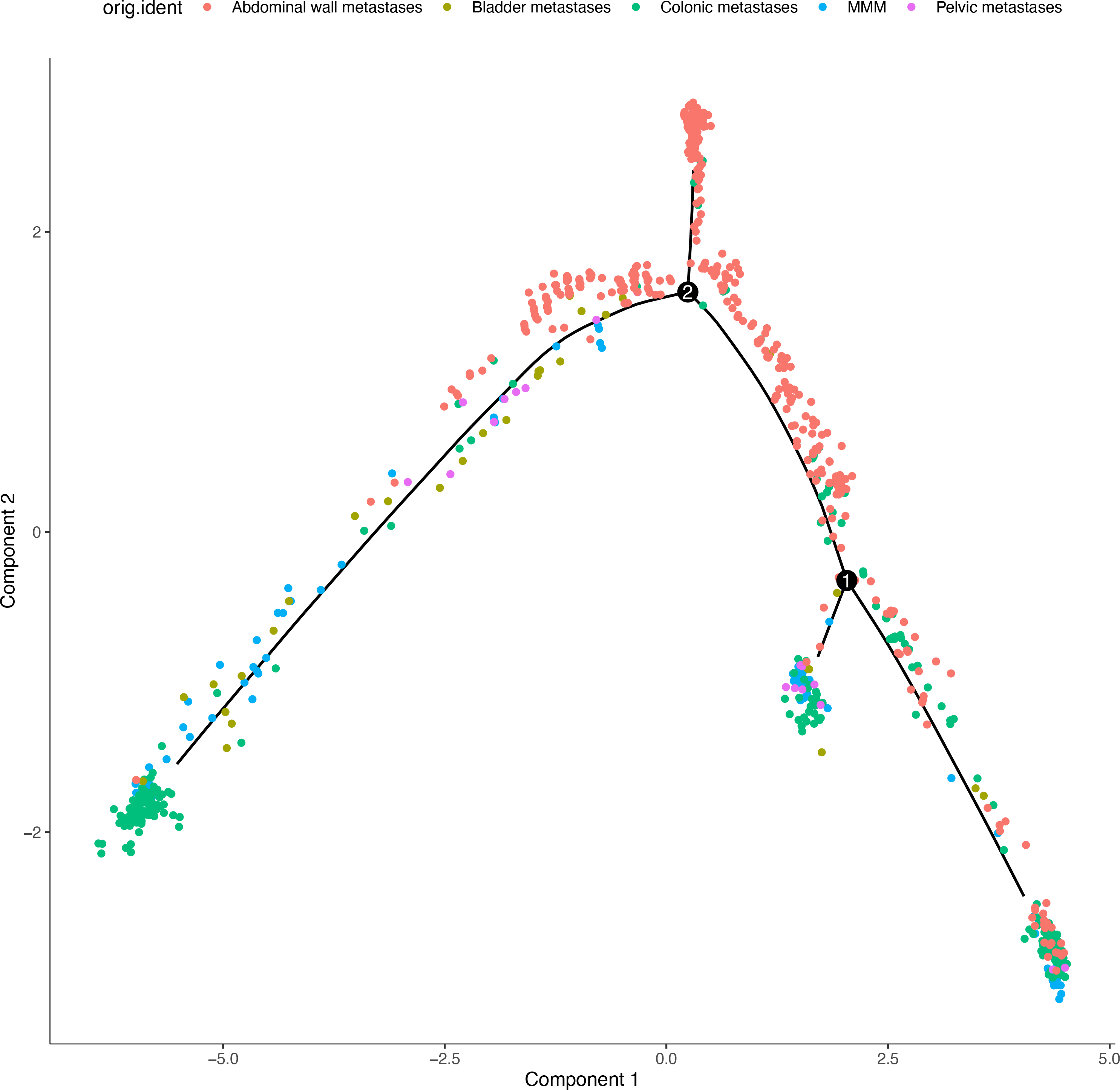

Supplement: Supplementary Figure 2 — B cell distribution across pseudotime in primary and four metastatic foci. [file Image2.tif]

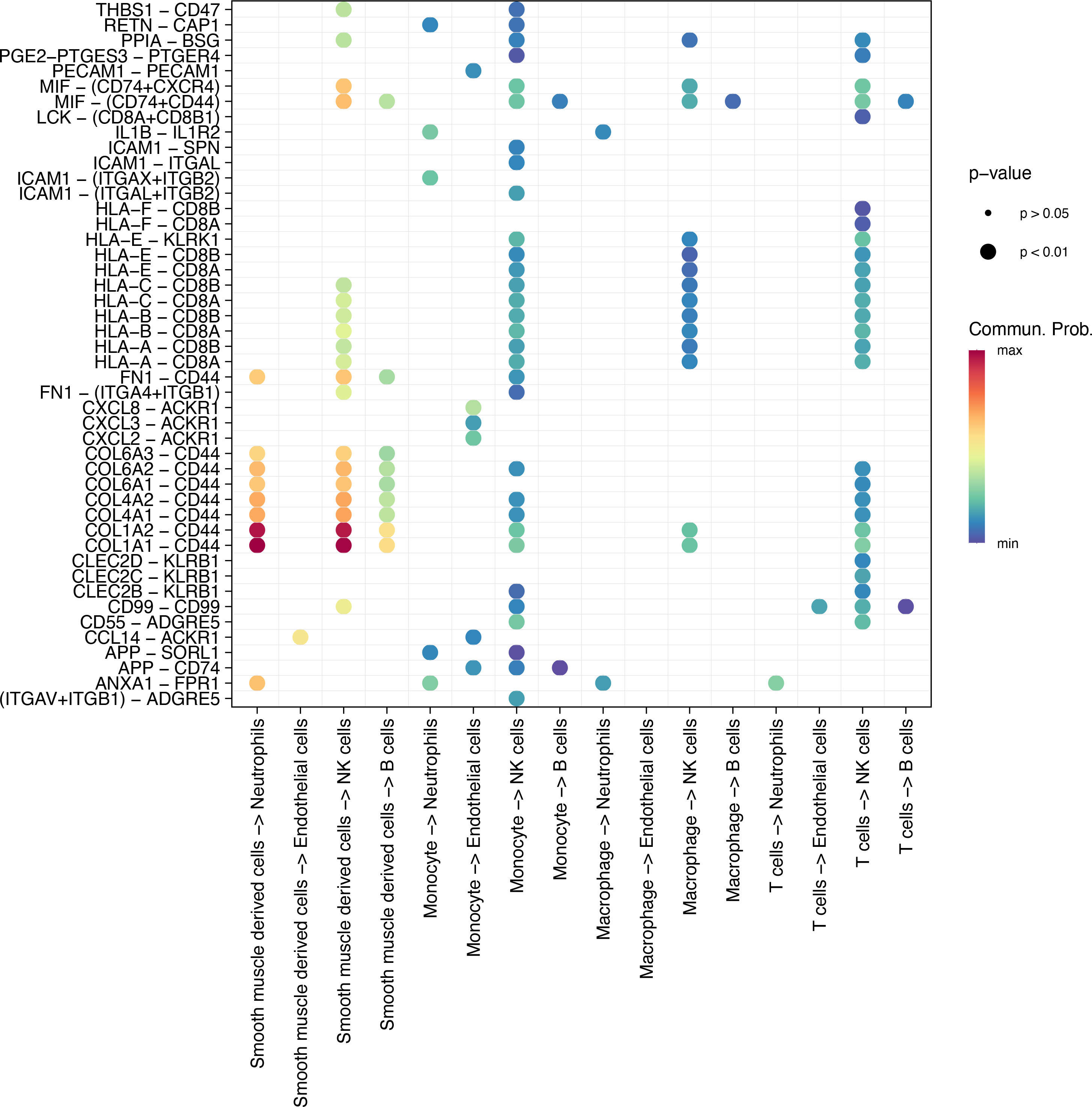

Supplement: Supplementary Figure 3 — Intercellular interactions among eight cell types with key receptor-ligand pair identification. [file Image3.tif]

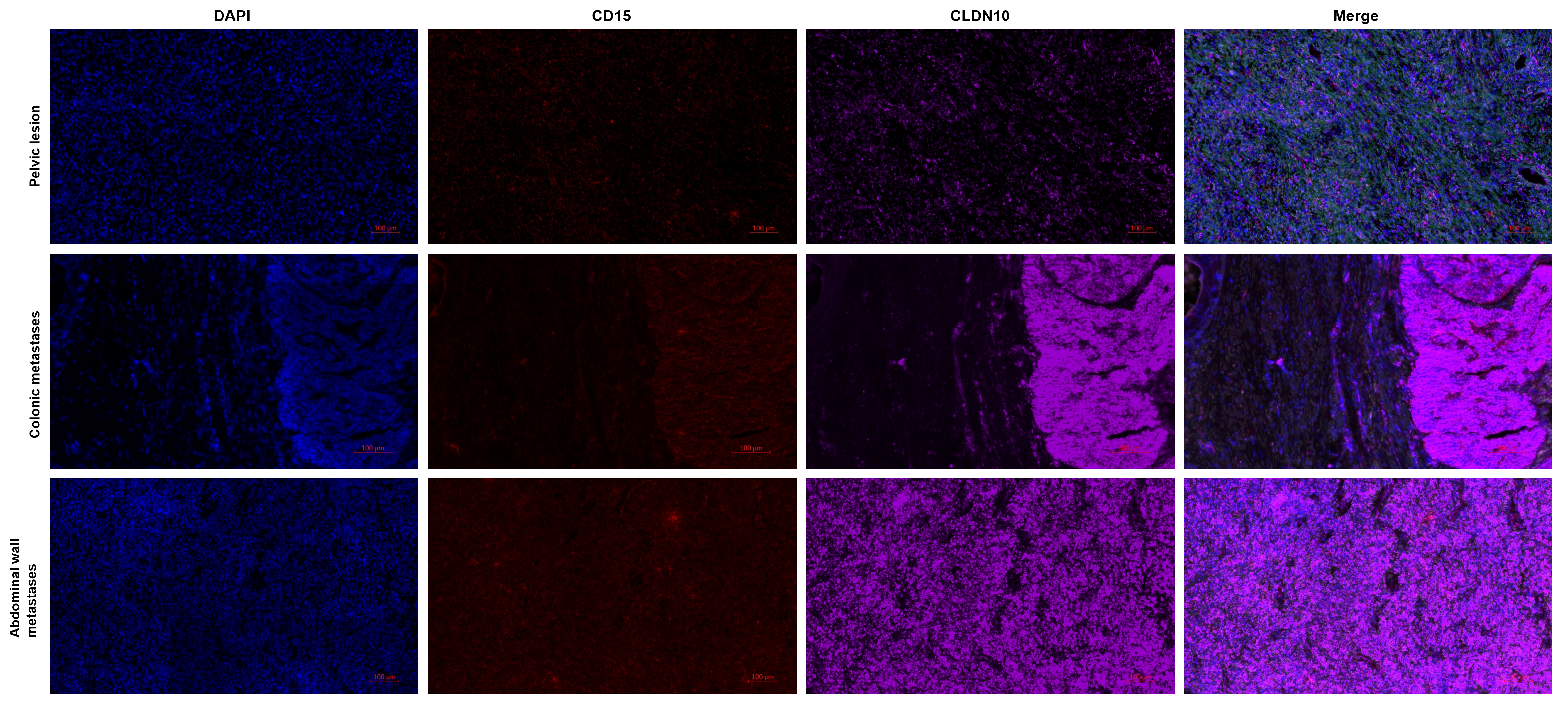

Supplement: Supplementary Figure 4 — Critical signaling pathways identified through cellular communication analysis. (A) Significant upregulation of CXCL8-ACKR1 signaling in monocyte-endothelial cell interactions. (B) Interaction strength for CXCL2-ACKR1 between monocytes and endothelial cells. (C) ICAM signaling network activity across all eight cell types. (D) ICAM1-(ITGAX+ITGB2) mediated communication within the cellular cohort. (E) THBS signaling pathway network among the characterized cell populations. [file Image4.tif]
